# Variations in Root and Canal Morphology of Mandibular First Molars: A Retrospective Cone-Beam Computed Tomography Study in a Saudi Subpopulation

**DOI:** 10.3390/jcm14238355

**Published:** 2025-11-24

**Authors:** Obadah Austah, Waad Ali, Amani Althebaiti, Mohammed Saleh Albati, Shatha Zahran, Mohammed Barayan, Abdullah Bokhary, Loai Alsofi

**Affiliations:** 1 Department of Endodontics, Faculty of Dentistry, King Abdulaziz University, P.O. Box 80299, Jeddah 21589, Saudi Arabia; sszzahran@kau.edu.sa (S.Z.); lalsofi@kau.edu.sa (L.A.); 2 Faculty of Dentistry, King Abdulaziz University, P.O. Box 80299, Jeddah 21589, Saudi Arabia; wali0025@stu.kau.edu.sa (W.A.); athibiti@stu.kau.edu.sa (A.A.); 3 General Practitioner, Apex Care Private Clinics, Qassim, Buraydah 52375, Saudi Arabia; mohammed.albuty@qudent.org; 4 Division of Oral and Maxillofacial Radiology, Department of Oral Diagnostic Sciences, Faculty of Dentistry, King Abdulaziz University, P.O. Box 80299, Jeddah 21589, Saudi Arabia; mbarayan@kau.edu.sa; 5 Department of Dental Public Health, Faculty of Dentistry, King Abdulaziz University, P.O. Box 80299, Jeddah 21589, Saudi Arabia; amabokhary@kau.edu.sa

**Keywords:** cone-beam computed tomography, mandibular first molar, root canal anatomy, middle mesial canal, Saudi Arabia

## Abstract

**Background/Objectives**: Successful endodontic treatment relies on a precise understanding of root canal morphology and effective disinfection of the entire canal system. Anatomical variations, such as additional canals, canal merging, and intercanal communications, may affect clinical outcomes. This retrospective CBCT study evaluated the root and canal morphology of mandibular first molars in a Saudi population, with specific focus on the prevalence and configuration patterns of middle mesial canals (MMCs) and isthmuses in the mesial root. **Methods**: A total of 552 CBCT scans taken between 2017 and 2021 were screened. After applying strict eligibility criteria, 167 scans from Saudi patients containing fully erupted mandibular first molars with completely formed apices were included, yielding 322 teeth. Teeth with prior root canal treatment or distorted scans were excluded. Root canal configurations were classified according to Weine’s system. The presence of MMCs, isthmuses, intercanal distance, and bilateral symmetry was recorded. Descriptive and comparative analyses were performed using Fisher’s exact test, chi-square test, Kruskal–Wallis test, and McNemar’s test. **Results**: Most mandibular first molars (75.5%) had two roots, whereas 24.5% exhibited two distinct distal roots. Mesial roots predominantly demonstrated Weine type II (50.9%) and type III (48.1%) configurations, while distal roots were mainly type I (69.3%). MMCs were rare (0.98%). Isthmuses were highly prevalent (82%) and occurred significantly more often in type III mesial roots (72.3%) compared with type II (1.2%) (*p* < 0.0001). Bilateral symmetry was substantial in both mesial and distal roots (86.8% and 88.4%, respectively). **Conclusions**: Middle mesial canals were uncommon, whereas isthmuses were frequently observed in the mesial roots of mandibular first molars among Saudi individuals. The high prevalence of isthmuses underscores the need to anticipate intercanal communications during endodontic treatment. CBCT evaluation enhances detection of such complex anatomical features and supports improved diagnostic accuracy and treatment planning.

## 1. Introduction

Endodontic treatment aims to prevent or treat apical periodontitis by thoroughly cleaning, shaping, and disinfecting the entire root canal system [1]. Technical deficiencies during this process can result in the persistence of bacteria within the canal system, potentially leading to treatment failure [2]. Thus, practitioners must possess adequate knowledge of pulp and root canal morphology, particularly for the lower first molar, as it is commonly associated with early carious involvement and subsequent need for root canal treatment [3]. Furthermore, investigations on outcomes have consistently demonstrated a decreased success rate in teeth characterized by intricate anatomical features [4], with a particular emphasis on mandibular molars [5,6].

Traditionally, mandibular first molars have been described as two-rooted molars with three canals, with two canals in the mesial root and one canal in the distal root. However, numerous studies have reported considerable variation in canal morphology and the number of roots, which appears to be strongly correlated with ethnic background and age, suggesting a genetic influence on these variations [7,8,9,10,11,12,13,14,15].

The mesial root of mandibular first molars presents a particularly complex anatomy, with a higher prevalence of isthmus and complex canal configurations [7,14,15]. Numerous studies have been conducted across diverse population groups to investigate the prevalence of the middle mesial canal and the anatomical characteristics of the mesial root in mandibular first molars, utilizing various methodologies, including root canal staining and tooth clearing [12], conventional and digital radiography [9], and radiographic assessment enhanced with contrast medium [16]. However, advanced modalities such as cone-beam computed tomography (CBCT) provide superior three-dimensional visualization, allowing more reliable identification of atypical anatomy, including the presence of middle mesial canals (MMCs) [17].

According to a systematic review by Bansal et al., the prevalence of MMCs in various populations ranged between 0.26% and 53.8% [18]. When guided troughing was performed using a dental operating microscope, MMCs were found in 46.2% of first molars [19]. Using CBCT, the prevalence of MMCs was 9.6% in the northwest Chinese population [20]. In the Middle East, the prevalence of three canals in the mesial root was found to be 17.5% [21]; in the Turkish population, the prevalence was 1.79% [22]. In the Saudi population specifically, the reported incidence of a middle mesial canal is generally low, ranging from 0.7% to 4.2% [23,24]. However, it is worth noting that there are exceptions to this trend, as evidenced by a CBCT study that reported a higher incidence of 18.2% [25]. Importantly, many of these studies did not specify nationality or ethnic distribution, which may contribute to variability due to underlying regional and genetic diversity.

The classification of root canal configurations has played a fundamental role in understanding internal root morphology and guiding endodontic management. Weine et al. (1969) [26] were the first to propose a systematic classification, identifying four major canal patterns within a single root: Type I (1-1), a single canal extending from chamber to apex; Type II (2-1), two canals merging into one; Type III (2-2), two separate canals running from chamber to apex; and Type IV (1-2), a single canal dividing into two apical branches. Later, Vertucci (1984) expanded this framework into eight configurations, describing additional canal divisions, rejoinings, and accessory pathways. Despite this refinement [27]. Despite Vertucci’s refinement, Weine’s simpler system remains widely used in CBCT-based studies due to its clarity, reproducibility, and ability to reliably describe major canal pathways. Notably, Weine Types II and III correspond closely to Vertucci Types II and IV, respectively, which are the most common morphologies in mesial roots of mandibular molars.

Considering the limited data on mandibular first molar morphology among Saudi individuals, particularly in the western region, this retrospective CBCT-based study was designed to characterize root and canal anatomy within a clearly defined Saudi subpopulation. The Weine classification was adopted for consistency and clinical interpretability [8,26]. Additionally, the study aimed to determine the prevalence and distribution of MMCs and isthmuses within mesial roots. We hypothesized that MMC prevalence would be low, whereas isthmuses would be highly common, consistent with previous regional trends. This study provides a novel contribution by analyzing a well-defined Saudi cohort using standardized CBCT criteria, offering data directly relevant to endodontic treatment planning and regional anatomical variation.

## 2. Materials and Methods

### Study Design and Ethical Approval

This retrospective observational study analyzed CBCT scans retrieved from the Oral and Maxillofacial Radiology Department at the Faculty of Dentistry, King Abdulaziz University, Jeddah, Saudi Arabia. Ethical approval was granted by the institutional Research Ethics Committee (Reference No. 151-12-20). All CBCT records were anonymized prior to analysis, and the study followed the Declaration of Helsinki and institutional research guidelines.

**Figure 1:**
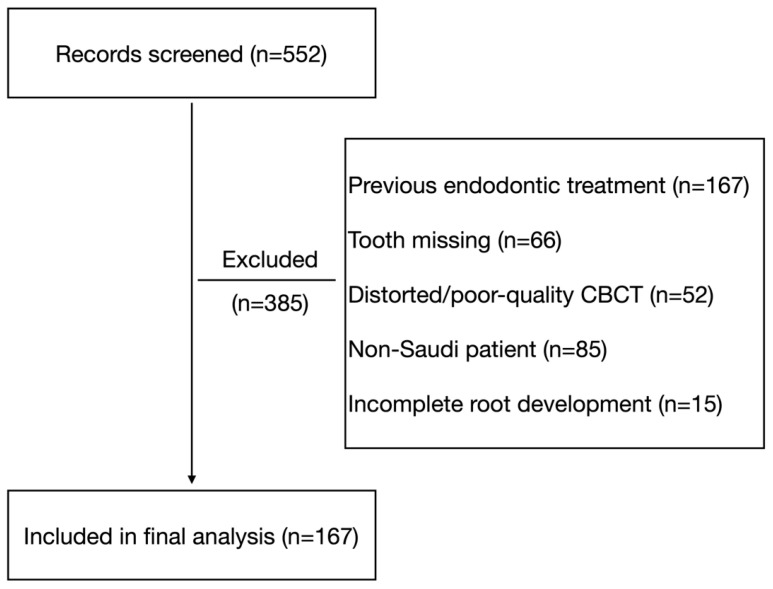
Flowchart of study sample selection showing 552 CBCT scans screened: 385 excluded and 167 included for analysis.

All CBCT records obtained for various diagnostic purposes acquired between 2017 and 2021 were screened for eligibility. A total of 552 scans were initially reviewed, and eligible scans were identified consecutively to avoid sampling bias (Figure 1). Only one scan per patient was included. Both mandibular first molars were evaluated when present, and a tooth-level analytic approach was used. The sample size was determined based on the average of previous CBCT studies that investigated mandibular molars in different populations [9,14,25].

## 3. Eligibility Criteria

Inclusion criteria
  Saudi patients aged ≥ 10 years.
  Fully erupted mandibular first molars.
  Completely formed root apices.
  CBCT scans with diagnostic image quality.
Exclusion criteria
  Previous root canal treatment.
  Large metallic restorations or crowns causing scatter.
  Calcified canals or internal/external resorption.
  Incomplete root formation.
  Motion artifacts or distorted CBCT images.

Scans in which only one mandibular first molar was present were excluded from the bilateral symmetry analysis.

### 3.1. CBCT Acquisition Parameters

All scans were acquired using an iCAT scanner (Imaging Sciences International, Hatfield, PA, USA). The scanning parameters were standardized for all patients: field of view (FOV) of 8 × 8 cm, voxel size of 125 μm, 90 kVp, 7 mA, and exposure time of 26.9 s. The reconstructed images were analyzed using OnDemand3D™ Imaging Software version 1.0.11 (Cybermed, Seoul, South Korea). Contrast and brightness were adjusted to ensure optimal visualization.

### 3.2. CBCT Image Processing and Root Alignment Protocol

To standardize comparisons, each mandibular first molar was reoriented along its long axis using multiplanar reconstruction tools. The long axis was first aligned in the sagittal plane, followed by refinement in coronal and axial planes until a uniform trajectory from the chamber floor to the apex was achieved. Axial images were evaluated at 0.125 mm intervals. Each tooth was examined sequentially from the orifice level to the apex using axial, sagittal, and coronal planes to confirm canal continuity, merging patterns, and root morphology.

### 3.3. Definitions and Criteria

#### 3.3.1. Canal Configuration

**Figure 2:**
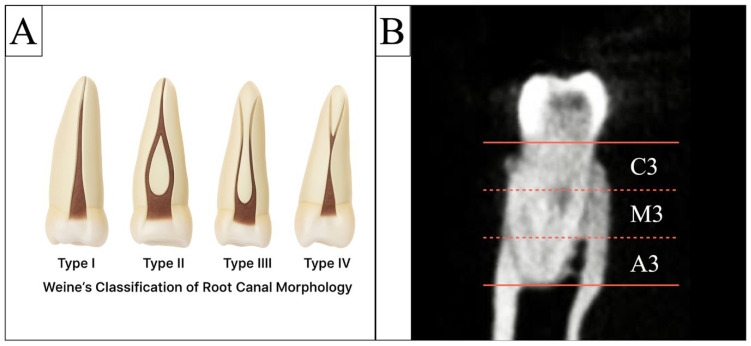
(**A**) Schematic illustration of Weine’s root canal configuration classification, demonstrating Type I (single canal), Type II (two canals merging into one), Type III (two separate canals), and Type IV (one canal dividing into two). (**B**) Representative coronal CBCT slice of a mandibular first molar illustrating the three isthmus levels evaluated in the mesial root: coronal third (C3), middle third (M3), and apical third (A3). The isthmus is visualized as a ribbon-shaped communication between the mesiobuccal and mesiolingual canals.

Canal morphology was categorized using Weine’s classification, applied separately to mesial and distal roots (Figure 2A) [8].

#### 3.3.2. Middle Mesial Canal (MMC)

**Figure 3:**
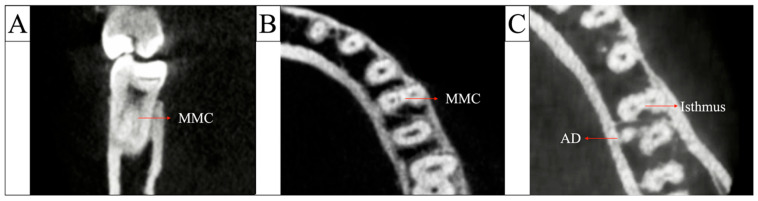
(**A**) Coronal CBCT slice showing a distinct middle mesial canal (MMC) between the MB and ML canals. (**B**) Axial view confirming the MMC as a separate canal within the mesial root. (**C**) Axial section demonstrating a mesial root isthmus and the presence of an accessory distal root (AD) compared with the main distal root.

An MMC was defined as a distinct canal located between the mesiobuccal (MB) and mesiolingual (ML) canals possessing the following characteristics (Figure 3A,B):

Visibility in ≥3 consecutive axial slices (0.125 mm intervals).

Confirmation on sagittal or coronal slices to verify continuity or merging toward either the MB or ML canal.

A canal that merged apically with either MB or ML was still classified as an MMC if criteria 1 and 2 were satisfied.

#### 3.3.3. Isthmus Classification

An isthmus was defined as a thin, ribbon-shaped connection between MB and ML canals (Figure 3C). Isthmuses were classified by their most coronal level (Figure 2B):

C3: coronal third;
M3: middle third;
A3: apical third.

Distal Root and Accessory Distal Root Criteria.

For teeth presenting with two distal roots, only the main distal root, defined as the larger, more centrally positioned root following the primary distal canal trajectory, was included in configuration analysis (Figure 3C). The accessory distal root, which consistently exhibited a single canal (Weine Type I), was recorded qualitatively but excluded from prevalence calculations to avoid duplicating root-level anatomy.

#### 3.3.4. Intercanal Distance

The distance between MB and ML canal orifices was measured in mm on the axial slice at the pulp chamber floor level.

### 3.4. Examiner Calibration and Reliability Analysis

Three calibrated evaluators—an endodontic resident, an endodontist, and an oral and maxillofacial radiologist—independently examined all CBCT scans under standardized viewing conditions using a 27-inch medical-grade monitor in dim lighting. All examiners were blinded to patient demographic information, including age and sex, to minimize interpretation bias; however, complete blinding to tooth side was not feasible due to inherent anatomical orientation. To assess calibration, twenty randomly selected scans were re-evaluated after four weeks. Inter-examiner and intra-examiner reliability were calculated separately for MMC detection, isthmus identification, and Weine canal configuration, yielding inter-examiner κ values ranging from 0.86 to 0.92 and intra-examiner κ values ranging from 0.87 to 0.94, indicating excellent agreement across all evaluated variables. Discrepant assessments were resolved by consensus review, ensuring consistency and uniform interpretation throughout the study.

### 3.5. Statistical Analysis

All demographic and radiographic data were recorded in Microsoft Excel (Version 16.7, Microsoft Corporation, Redmond, WA, USA) and analyzed using SAS^®^ OnDemand for Academics version 9.4 (SAS Institute Inc., Cary, NC, USA). Descriptive statistics summarized root type number, Weine canal configurations, the prevalence of isthmuses, and the location of the isthmuses in the roots. Intercanal distances were compared using the Kruskal–Wallis test after inspection of normality (Shapiro–Wilk and Q-Q plots). Categorical differences in Weine canal configuration distributions by root type, sex, and age group were evaluated with Fisher’s exact test and chi-squared test where appropriate. A multinomial regression analysis model was conducted on each root type separately to assess the canal configurations by gender, adjusting for age. Symmetry between contralateral teeth was assessed with McNemar’s test for mesial roots and Bowker’s test of symmetry for distal roots, and agreement was quantified using unweighted Cohen’s κ. The *p*-value <0.05 was considered statistically significant.

## 4. Results

Of the 552 CBCT scans screened, 385 were excluded for various reasons (Figure 1). A total of 167 CBCT scans fulfilled all inclusion criteria and were included for analysis, providing 322 mandibular first molars for morphological analysis. A total of 723 roots were evaluated for canal morphology (322 mesial and 401 distal). The study sample comprised 73 males (43.7%) and 94 females (56.3%), with a mean age of 31.0 ± 11.8 years (range 18–72 years).

Most mandibular first molars (243) showed two main roots (mesial and distal), accounting for 75.5% of the total examined teeth. However, 79 teeth (24.5%) exhibited two separate distal roots, leading to a more complex root structure in the distal area. Canal configuration analyses were therefore conducted separately for all mesial and distal roots, including those in teeth with additional distal roots.

As summarized in Table 1, Fisher’s exact test demonstrated a significant difference in canal configuration between root types (*p* < 0.001). Mesial roots were almost entirely Weine II (50.9%) and III (48.1%), with only 0.9% of type I, and none were type IV. Main distal roots were predominantly Weine I (69.3%), with smaller proportions of types II (20.2%), III (6.2%), and IV (4.3%).

In Table 2, Fisher’s exact tests demonstrated that canal configuration distributions varied by both sex and age. For mesial roots, males showed a higher share of Weine III (53.5%) and a lower share of II (45.8%) compared with females (type III = 43.9%, type II = 55.0%; *p* = 0.009). For main distal roots, males had more type IV than females (7.0% vs. 2.2%), and with respect to type I, 67.6% compared to 70.6% in females (*p* = 0.001). Age effects were also significant (*p* < 0.001) for both root types. In mesial roots, the balance between II and III in the youngest group (≤20: type II = 51.0%, type III = 49.0%) shifted toward more type II in middle age (51–60: type II = 71.4%, type III = 28.6%). In distal roots, type I dominated in the youngest group (≤20: 86.3%) but decreased in the oldest group (≥61: 25.0%), whereas types II and III each reached 37.5%. Overall, these patterns indicate sex- and age-related differences in canal configuration, with distal roots showing a clear age-related shift away from simple type I anatomy at the descriptive level. However, in multinomial regression models fitted separately for mesial and distal roots and adjusted for age, the association between sex and canal configuration did not show statistical significance.

In the mesial roots, the mean intercanal distance showed no significant difference between Weine type II and III configurations (Kruskal–Wallis, *p* = 0.435). Isthmus presence, however, was significantly more frequent in Weine type III (88.4%) compared with type II (75.6%; *p* = 0.003), yielding an overall prevalence of 82%. With respect to the level of occurrence, apical third (A3) isthmuses were markedly more common in Weine type III (72.3%) than in type II (1.2%; *p* < 0.001), whereas middle (M3) (51.8% vs. 58.1%; *p* = 0.263) and coronal (C3) (54.9% vs. 54.8%; *p* = 0.994) levels showed no significant differences between the two configurations (Table 3). Representative CBCT images illustrating isthmus classification and section levels (C3, M3, A3) are shown in Figure 2B.

Among distal roots, Weine type I predominated in the overall sample (90.5%, 220/243), with lower frequencies of type II (6.6%), type III (2.1%), and type IV (0.8%). In contrast, for two-distal-root molars (main distal root only), complex configurations were markedly more common (type II (62.0%), type III (19.0%), and type IV (15.2%)), while type I was rare (3.8%) (Table 4). Fisher’s exact test confirmed a significant difference in configuration distributions between groups (*p* < 0.001). These findings indicate that, although simple single-canal anatomy (type I) predominates across distal roots overall, the main distal root in 3-rooted teeth more frequently exhibits multicanal (types II–IV) patterns, underscoring the need for thorough preoperative imaging to anticipate canal complexity in such cases.

Symmetry between contralateral mandibular first molars (right vs. left) was high at the root level. Mesial roots matched in 86.8% of pairs (132/152) with unweighted Cohen’s κ = 0.74, *p* < 0.001. The McNemar’s test showed no left–right asymmetry (*p* = 0.371). Distal roots matched in 88.4% (137/155) with unweighted κ = 0.76, *p* < 0.001. The Bowker’s test likewise indicated no asymmetry (*p* = 0.423) (Table 5). Together, these findings support substantial bilateral morphological concordance with balanced discordances and no systematic side preference.

At the whole-tooth level, symmetry was 76.1% (118/155), whereas by sex, symmetry was comparable: 76.7% in females vs. 75.4% in males (χ^2^ *p* = 0.852). Across age groups, symmetry ranged from 62.5% (41–50) to 87.0% (31–40). The chi-squared test revealed no significant differences by age (*p* = 0.325) (Table 6).

## 5. Discussion

In the present study, CBCT evaluation of mandibular first molars in a Saudi subpopulation demonstrated that complex mesial anatomy was common, primarily in the form of intercanal communications, whereas a distinct middle mesial canal (MMC) was rare (0.98%). Although previous literature has reported considerable variability in MMC prevalence across populations [10,15,19,21,28], our findings indicate that the dominant anatomical feature in this cohort was the high frequency of isthmuses (82%) rather than the presence of an additional canal. This distinction is important because it underscores that, within this population, morphological complexity is expressed mainly through fins and interconnections rather than discrete, separate canals. Accordingly, clinical emphasis should be placed on recognizing these intercanal communications, which may influence debridement challenges, rather than assuming the routine presence of an MMC.

The present study demonstrated a slightly lower prevalence of middle mesial canals (MMCs) compared with earlier Saudi reports, where rates ranged from 1.3% to 4.2% [23,24]. This variation may reflect regional demographic differences, as the western region, where the current sample originated, is characterized by greater ethnic diversity. This demographic heterogeneity likely accounts for subtle differences from studies conducted in the central region [23,24]. A more recent report by Madfa et al. found MMC prevalence rates of 2.6% and 1.6% in left and right mandibular first molars, respectively [29], which are higher than the 0.98% observed here. The comparatively low MMC frequency supports the influence of population-specific and genetic factors and highlights the need for regional CBCT-based morphological investigations. When compared with international data showing substantially higher MMC rates [19,25], the current findings further underscore that root canal configuration varies across ethnic groups and should be interpreted within appropriate demographic contexts.

International comparisons similarly demonstrate substantial ethnic variability in MMC prevalence. Wang et al. (2025) reported a 9.6% MMC rate in a northwestern Chinese population, much higher than the Saudi prevalence, suggesting that differences in intercanal spacing and morphometric patterns may facilitate additional canal formation [20]. In contrast, Şendişçi Gök et al. (2025) documented low frequencies of accessory roots in Turkish mandibular molars, findings that closely resemble the Saudi pattern [30]. Although MMCs remain uncommon in Middle Eastern populations, their occasional presence has important clinical implications, reinforcing the value of CBCT in preoperative assessment. The superior three-dimensional detail of CBCT compared with conventional radiography likely explains part of the variability in reported MMC rates across studies [19,25].

Reports from other populations also show age-related variability in MMC expression. Some studies observed a higher MMC occurrence in middle-aged individuals [31], suggesting that internal canal morphology may change over time, although the mechanisms remain unclear. Within Saudi Arabia, national data indicate that anatomical variation is influenced by population heterogeneity and regional diversity [24]. Comparable international findings, such as those reported by Silva et al. in a Brazilian cohort [14], demonstrate that population structure contributes significantly to morphological variation in mandibular molars. Together, these observations reinforce that demographic differences can shape canal configurations and support the use of individualized radiographic assessment during endodontic planning.

In the current study, mesial roots predominantly exhibited Weine type II (50.9%) and type III (48.1%) configurations, whereas distal roots most frequently presented as type I (69.3%). Teeth with two distinct distal roots (24.5%) demonstrated noticeably greater morphological complexity, with the main distal root displaying a higher proportion of multicanal configurations (Weine types II–IV). These observations closely align with the national systematic review by Mashyakhy et al. (2022), which reported that Saudi mandibular first molars primarily exhibit two roots with mesial configurations corresponding to Vertucci types II and IV and distal configurations largely classified as type I [24]. The present findings are further supported by earlier Saudi and international investigations [12,24,28,32,33]. Consistent with previous reports, the presence of an additional distal root was associated with increased canal variability [21,24], mirroring global observations that duplicated distal roots often exhibit more complex internal anatomy [10,11,28].

The present study identified statistically significant associations between canal configuration and both sex and age; however, these findings should be interpreted with caution, as they arose from univariate analyses rather than adjusted multivariable models. Descriptively, males demonstrated a slightly higher proportion of complex configurations (Weine types III–IV) in both mesial and distal roots, while females more commonly exhibited simpler patterns (types I–II). Similar tendencies have been noted in previous CBCT-based studies, although several large Saudi investigations reported no significant gender differences in root or canal number [34]. Likewise, Pattanshetti et al. [9] observed only non-significant trends toward greater multicanal incidence in males. These collective findings indicate that potential sex-related anatomical differences remain inconsistent across the literature and should not be inferred as causal without robust adjusted analyses.

Regarding age, a progressive shift toward simpler distal configurations in older individuals was noted, consistent with prior CBCT and micro-CT reports showing reduced canal complexity with age [28,35]. These findings may reflect general age-related morphological changes described in the literature, yet, given the absence of adjusted analyses in the present study, they should not be interpreted as evidence of causal biological mechanisms. Instead, the results highlight demographic patterns that warrant further investigation using multivariable models in future studies.

The intercanal distance between the mesiobuccal and mesiolingual orifices ranged from 1.0 to 3.4 mm (mean ≈ 2 mm). Although this measurement was not associated with MMC occurrence, it remains clinically relevant because wider inter-orifice spacing has been linked to a greater likelihood of intercanal communications. In the present study, isthmuses were identified in 82% of mesial roots-consistent with earlier CBCT, micro-CT, and histologic reports showing rates between 80% and 100% [7,36,37,38]. Importantly, the apical third demonstrated the highest isthmus prevalence in Weine type III configurations, whereas type II roots showed more coronal and middle third connections. This apical predominance aligns with Mannocci et al. [36], who reported frequent isthmuses 3–5 mm from the apex, and with micro-CT studies demonstrating dense apical interconnections [7,36]. Additional CBCT studies, including Al-Habib et al. [34] and others [39,40], further support the high anatomical complexity of this region.

From a clinical perspective, isthmuses present a major challenge because their narrow and irregular extensions frequently harbor debris and bacterial biofilms that are difficult to eliminate using standard instrumentation [41,42]. Although the present study did not assess treatment outcomes or specific irrigation strategies, the high prevalence of isthmuses observed reinforces the need for careful canal exploration, adequate visualization, and thorough cleaning during root canal therapy. Prior research has shown that complex canal systems may benefit from enhanced irrigant penetration, including activation-based methods [43,44,45,46], and that teeth with Weine type II configurations, often resembling isthmus-like anatomy, may show delayed healing [4]. These external findings highlight why recognizing isthmus morphology is clinically relevant, even though the current study was not designed to evaluate clinical protocols.

Moreover, symmetry analysis demonstrated a high degree of concordance (86.6–88.4%) in canal configurations between right and left mandibular first molars, consistent with previous reports [32,47]. Using CBCT and the Weine classification, the present study provided a comprehensive three-dimensional assessment of mandibular molar canal morphology. Compared with conventional two-dimensional radiography, CBCT offers superior accuracy for identifying additional canals, isthmuses, and merging patterns [10,40]. Furthermore, the classic radiographic work of Pineda and Kuttler, based on 7275 teeth, described frequent hidden curvatures, bifurcations, and off-center apical exits, features that are now more clearly visualized using CBCT. The high isthmus prevalence and multicanal configurations observed in the present study reinforce these foundational findings and underscore the inherent complexity of mandibular molar anatomy across populations and imaging modalities [48].

Despite its strengths, this study has several limitations. The retrospective use of pre-existing CBCT scans—originally acquired for various diagnostic purposes—may introduce selection bias. Furthermore, the sample was derived from a single institutional database, which restricts external validity and may not fully capture morphological variability across different Saudi regions. Although descriptive analyses suggested sex- and age-related differences in canal configuration, these patterns should be interpreted cautiously, as multivariable models adjusted for age and sex did not demonstrate statistically significant associations. The absence of broader adjusted analyses therefore limits the ability to draw causal inferences about demographic influences. Future multicenter investigations with standardized CBCT acquisition protocols, larger and more diverse samples, and fully adjusted analytical models are recommended to validate these findings and explore regional variation in mandibular molar morphology.

## 6. Conclusions

In conclusion, the present study demonstrated that the prevalence of a middle mesial canal in mandibular first molars among the Saudi population was exceptionally low (0.98%), whereas isthmuses were highly prevalent (approximately 82%), particularly within the mesial roots. Nearly one-quarter of examined teeth exhibited two distinct distal roots, and these teeth showed more complex distal canal configurations than single-rooted counterparts. Collectively, these findings highlight the substantial anatomical variability of mandibular first molars and underscore the importance of thorough preoperative imaging and careful clinical exploration during endodontic treatment. CBCT evaluation provides valuable diagnostic insight into canal morphology, including duplications, variations, and intercanal communications, that may be difficult to detect using conventional radiography. A detailed understanding of these morphological features is essential for optimizing cleaning, shaping, and obturation strategies, reducing the risk of missed canals, persistent infection, and postoperative complications.

### Clinical Significance

A comprehensive understanding of mandibular first molar morphology is essential for predictable endodontic outcomes. This study demonstrated a very low prevalence of middle mesial canals (0.98%) but a notably high incidence of isthmuses (82%) within the mesial root, underscoring the importance of anticipating intercanal communications rather than additional canals. The strong bilateral symmetry observed at both the root (86–88%) and whole-tooth (76%) levels suggests that the anatomy of one mandibular first molar can serve as a reliable reference for its contralateral counterpart. These morphological insights highlight the value of thorough preoperative assessment—particularly via CBCT when clinically justified—to improve canal localization, guide access design, and support more informed clinical decision-making. 

## Figures and Tables

**Table 1:** Statistical comparison of Weine’s canal configurations according to root type.

Root Type	Weine’s Canal Configurations				
	I	II	III	IV	*p*-Value *
Mesial Root	3	164	155	0	<0.001
n = 322	(0.9%)	(50.9%)	(48.1%)	(0.0%)	
Distal (Main) Root	223	65	20	14	
n = 322	(69.3%)	(20.2%)	(6.2%)	(4.3%)	

* Fisher’s exact test (*p* < 0.05 is considered significant).

**Table 2:** Distribution of Weine’s canal configurations in mesial and distal roots of mandibular first molars according to gender and age group.

Variable	Category	Mesial Root					Distal Main Root				
		I	II	III	IV	*p*-Value *	I	II	III	IV	*p*-Value *
Gender	Male	1 (0.7%)	65 (45.8%)	76 (53.5%)	0 (0.0%)	0.009	96 (67.6%)	28 (19.7%)	8 (5.6%)	10 (7.0%)	0.001
	Female	2 (1.1%)	99 (55.0%)	79 (43.9%)	0 (0.0%)		127 (70.6%)	37 (20.6%)	12 (6.6%)	4 (2.2%)	
Age	≤ 20	0 (0.0%)	26 (51.0%)	25 (49.0%)	0 (0.0%)	<0.001	44 (86.3%)	4 (7.8%)	0 (0.0%)	3 (5.9%)	<0.001
	21–30	3 (2.5%)	62 (51.2%)	56 (46.3%)	0 (0.0%)		80 (66.1%)	27 (22.3%)	8 (6.6%)	6 (5.0%)	
	31–40	0 (0.0%)	48 (50.0%)	48 (50.0%)	0 (0.0%)		62 (64.6%)	22 (22.9%)	7 (7.3%)	5 (5.2%)	
	41–50	0 (0.0%)	14 (43.8%)	18 (56.3%)	0 (0.0%)		24 (75.0%)	6 (18.8%)	2 (6.2%)	0 (0.0%)	
	51–60	0 (0.0%)	10 (71.4%)	4 (28.6%)	0 (0.0%)		11 (78.6%)	3 (21.4%)	0 (0.0%)	0 (0.0%)	
	≥61	0 (0.0%)	4 (50.0%)	4 (50.0%)	0 (0.0%)		2 (25.0%)	3 (37.5%)	3 (37.5%)	0 (0.0%)	

* Fisher’s exact test. (*p* < 0.05 is considered significant).

**Table 3:** Distribution of intercanal distance and isthmus characteristics in mesial roots of mandibular first molars according to Weine’s canal configuration.

Weine Type	n (Mesial Roots)	Intercanal Distance in mm (Mean ± SD)	Isthmus Present n (%)	Isthmus Level A3 n (%)	Isthmus Level M3 n (%)	Isthmus Level C3 n (%)
II	164	1.97 ± 0.43	124 (75.6%)	2 (1.2%)	85 (51.8%)	90 (54.9%)
III	155	1.93 ± 0.51	137 (88.4%)	112 (72.3%)	90 (58.1%)	85 (54.8%)
*p*-value		0.435 *	0.003 ^§^	<0.001 ^§^	0.263 ^§^	0.994 ^§^

* Kruskal–Wallis, ^§^ Chi-squared (χ^2^) test. (*p* < 0.05 is considered significant).

**Table 4:** Comparison of Weine’s canal configurations between all distal roots and those in teeth with two distinct distal roots.

Weine Type	All Distal Roots (n = 243)	Two-Distal-Root Molars (*n = 79, Main Distal Root Only*)	*p*-Value *
I	220 (90.5%)	3 (3.8%)	<0.001
II	16 (6.6%)	49 (62.0%)	
III	5 (2.1%)	15 (19.0%)	
IV	2 (0.8%)	12 (15.2%)	
Total	243 (100%)	79 (100%)	

* Fisher’s exact test. (*p* < 0.05 is considered significant).

**Table 5:** Root-level symmetry (Weine’s classification) between right and left molars.

Root Type	Paired (n)	Matching (n)	Agreement	Unweighted Cohen’s κ	*p*-Value
Mesial	152	132	86.8%	0.74	<0.001 *
Distal	155	137	88.4%	0.76	<0.001 ^§^

* McNemar’s test. ^§^ Bowker’s test. (*p* < 0.05 is considered significant).

**Table 6:** Whole-tooth symmetry according to gender and age.

Variable	Category	Total n	Symmetry Present n (%)	*p*-Value *
Overall	All	155	118 (76.1%)	0.852
Gender	Female	86	66 (76.7%)	
	Male	69	52 (75.4%)	
Age group	≤20	25	17 (68.0%)	0.325
	21–30	58	44 (75.9%)	
	31–40	46	40 (87.0%)	
	41–50	16	10 (62.5%)	
	51–60	6	4 (66.7%)	
	≥60	4	3 (75.0%)	

* Chi-squared (χ^2^) test. (*p* < 0.05 is considered significant).

## Data Availability

The datasets generated and/or analyzed during the current study are available from the corresponding author on reasonable request. Due to participant confidentiality and institutional regulations, raw examiner-related data cannot be publicly shared; however, anonymized data underlying the findings of this article can be provided upon request.

## References

[1] European Society of Endodontology (2006). Quality guidelines for endodontic treatment: Consensus report of the European Society of Endodontology. Int. Endod. J..

[2] Nair P.N. (1997). Apical periodontitis: A dynamic encounter between root canal infection and host response. Periodontol. 2000.

[3] Berkovitz B.K., Holland G., Muxham B.J. (1992). Tooth morphology. Oral Anatomy Histology and Embryology.

[4] Azim A.A., Griggs J.A., Huang G.T. (2016). The Tennessee study: Factors affecting treatment outcome and healing time following nonsurgical root canal treatment. Int. Endod. J..

[5] Smith C.S., Setchell D.J., Harty F.J. (1993). Factors influencing the success of conventional root canal therapy--a five-year retrospective study. Int. Endod. J..

[6] Swartz D.B., Skidmore A.E., Griffin J.A. (1983). Twenty years of endodontic success and failure. J. Endod..

[7] Gu L., Wei X., Ling J., Huang X. (2009). A microcomputed tomographic study of canal isthmuses in the mesial root of mandibular first molars in a Chinese population. J. Endod..

[8] Weine F.S., Hayami S., Hata G., Toda T. (1999). Canal configuration of the mesiobuccal root of the maxillary first molar of a Japanese sub-population. Int. Endod. J..

[9] Pattanshetti N., Gaidhane M., Al Kandari A.M. (2008). Root and canal morphology of the mesiobuccal and distal roots of permanent first molars in a Kuwait population—A clinical study. Int. Endod. J..

[10] Kim S.Y., Yang S.E. (2012). Cone-beam computed tomography study of incidence of distolingual root and distance from distolingual canal to buccal cortical bone of mandibular first molars in a Korean population. J. Endod..

[11] Chandra S.S., Chandra S., Shankar P., Indira R. (2011). Prevalence of radix entomolaris in mandibular permanent first molars: A study in a South Indian population. Oral Surg. Oral Med. Oral Pathol. Oral Radiol. Endod..

[12] Alavi A.M., Opasanon A., Ng Y.L., Gulabivala K. (2002). Root and canal morphology of Thai maxillary molars. Int. Endod. J..

[13] Awawdeh L., Abdullah H., Al-Qudah A. (2008). Root form and canal morphology of Jordanian maxillary first premolars. J. Endod..

[14] Silva E.J.N.L., Nejaim Y., Silva A.V., Haiter-Neto F., Cohenca N. (2013). Evaluation of root canal configuration of mandibular molars in a Brazilian population by using cone-beam computed tomography: An in vivo study. J. Endod..

[15] Valencia de Pablo Ó., Estevez R., Peix M., Heilborn C., Cohenca N. (2010). Root anatomy and canal configuration of the permanent mandibular first molar: A systematic review. J. Endod..

[16] Naoum H., Love R., Chandler N., Herbison P. (2003). Effect of X-ray beam angulation and intraradicular contrast medium on radiographic interpretation of lower first molar root canal anatomy. Int. Endod. J..

[17] Cotton T.P., Geisler T.M., Holden D.T., Schwartz S.A., Schindler W.G. (2007). Endodontic applications of cone-beam volumetric tomography. J. Endod..

[18] Bansal R., Hegde S., Astekar M. (2018). Morphology and prevalence of middle canals in the mandibular molars: A systematic review. J. Oral Maxillofac. Pathol..

[19] Azim A.A., Deutsch A.S., Solomon C.S. (2015). Prevalence of middle mesial canals in mandibular molars after guided troughing under high magnification: An in vivo investigation. J. Endod..

[20] Wang D., Wang R., Xu H., Zhang Q., Guo Y. (2025). Prevalence and morphology of middle mesial canals in mandibular first molars and their relationship with anatomical aspects of the mesial root: A CBCT analysis. BMC Oral Health.

[21] Al Shehadat S., Waheb S., Al Bayatti S.W., Kheder W., Khalaf K., Murray C.A. (2019). Cone beam computed tomography analysis of root and root canal morphology of first permanent lower molars in a Middle East subpopulation. J. Int. Soc. Prev. Community Dent..

[22] Qiao X., Zhu H., Yan Y., Li J., Ren J., Gao Y., Zou L. (2020). Prevalence of middle mesial canal and radix entomolaris of mandibular first permanent molars in a western Chinese population: An in vivo cone-beam computed tomographic study. BMC Oral Health.

[23] Aldosimani M.A., Althumairy R.I., Alzahrani A., Aljarbou F.A., Alkatheeri M.S., AlGhizzi M.A., Abughosh T.K. (2021). The mid-mesial canal prevalence in mandibular molars of a Saudi population: A cone-beam computed tomography study. Saudi Dent. J..

[24] Mashyakhy M., AlTuwaijri N., Alessa R., Alazzam N., Alotaibi B., Almutairi R., Alroomy R., Thota G., Melha A.A., Alkahtany M.F. (2022). Anatomical Evaluation of Root and Root Canal Morphology of Permanent Mandibular Dentition among the Saudi Arabian Population: A Systematic Review. BioMed Res. Int..

[25] Srivastava S., Alrogaibah N.A., Aljarbou G. (2018). Cone-beam computed tomographic analysis of middle mesial canals and isthmus in mesial roots of mandibular first molars-prevalence and related factors. J. Conserv. Dent. Endod..

[26] Weine F.S., Healey H.J., Gerstein H., Evanson L. (1969). Canal configuration in the mesiobuccal root of the maxillary first molar and its endodontic significance. Oral Surg. Oral Med. Oral Pathol..

[27] Vertucci F.J. (1984). Root canal anatomy of the human permanent teeth. Oral Surg. Oral Med. Oral Pathol..

[28] Chen G., Yao H., Tong C. (2009). Investigation of the root canal configuration of mandibular first molars in a Taiwan Chinese population. Int. Endod. J..

[29] Madfa A.A., Alshammari A.F., Almagadawyi E., Al-Haddad A., Aledaili E.A. (2025). Prevalence of radix entomolaris and distolingual canals and their association with the incidence of middle mesial canals in mandibular first molars of a Saudi subpopulation. Sci. Rep..

[30] Şendişçi Gök R., Tercanlı H., Ekinci A. (2025). Evaluation of root and canal morphology of mandibular molar teeth by cone beam computed tomography: Cross-sectional study. BMC Oral Health.

[31] Bhatti U.A., Muhammad M., Javed M.Q., Sajid M. (2022). Frequency of middle mesial canal in mandibular first molars and its association with various anatomic variables. Aust. Endod. J..

[32] Huang C.-C., Chang Y.-C., Chuang M.-C., Lai T.-M., Lai J.-Y., Lee B.-S., Lin C.-P. (2010). Evaluation of root and canal systems of mandibular first molars in Taiwanese individuals using cone-beam computed tomography. J. Formos. Med. Assoc..

[33] Vertucci F., Williams R. (1974). Root canal anatomy of the mandibular first molar. J. N. J. Dent. Assoc..

[34] Al-Habib M.A., Almarzouki S., Alsulaiman M., Alsofi L. (2024). Comprehensive Analysis of Mandibular First Molar Root and Canal Morphology in Saudi Patients Using Cone Beam Computed Tomography (CBCT). Med. Sci. Monit..

[35] Kim Y., Perinpanayagam H., Lee J.K., Yoo Y.J., Oh S., Gu Y., Lee S.P., Chang S.W., Lee W., Baek S.H. (2015). Comparison of mandibular first molar mesial root canal morphology using micro-computed tomography and clearing technique. Acta Odontol. Scand..

[36] Mannocci F., Peru M., Sherriff M., Cook R., Pitt Ford T.R. (2005). The isthmuses of the mesial root of mandibular molars: A micro-computed tomographic study. Int. Endod. J..

[37] Fan B., Pan Y., Gao Y., Fang F., Wu Q., Gutmann J.L. (2010). Three-dimensional morphologic analysis of isthmuses in the mesial roots of mandibular molars. J. Endod..

[38] Keles A., Keskin C. (2018). Deviations of mesial root canals of mandibular first molar teeth at the apical third: A micro–computed tomographic study. J. Endod..

[39] Teixeira F.B., Sano C.L., Gomes B.P.F.A., Zaia A.A., Ferraz C.C.R., Souza-Filho F.J. (2003). A preliminary in vitro study of the incidence and position of the root canal isthmus in maxillary and mandibular first molars. Int. Endod. J..

[40] Xu S., Dao J., Liu Z., Zhang Z., Lu Y., Zeng X. (2020). Cone-beam computed tomography investigation of middle mesial canals and isthmuses in mandibular first molars in a Chinese population. BMC Oral Health.

[41] Nair P.N., Henry S., Cano V., Vera J. (2005). Microbial status of apical root canal system of human mandibular first molars with primary apical periodontitis after “one-visit” endodontic treatment. Oral Surg. Oral Med. Oral Pathol..

[42] Siqueira J.F., Rôças I.N., Ricucci D. (2010). Biofilms in endodontic infection. Endod. Top..

[43] Gutarts R., Nusstein J., Reader A., Beck M. (2005). In vivo debridement efficacy of ultrasonic irrigation following hand-rotary instrumentation in human mandibular molars. J. Endod..

[44] Plotino G., Özyürek T., Gündoğar M., Uslu G., Pedullà E., Careddu R., Franco V. (2023). Efficacy of different irrigant activation devices in removing dentin debris from an artificial isthmus connecting curved canals. Aust. Endod. J..

[45] Rödig T., Koberg C., Baxter S., Konietschke F., Wiegand A., Rizk M. (2019). Micro-CT evaluation of sonically and ultrasonically activated irrigation on the removal of hard-tissue debris from isthmus-containing mesial root canal systems of mandibular molars. Int. Endod. J..

[46] Malentacca A., Uccioli U., Mannocci F., Bhuva B., Zangari D., Pulella C., Lajolo C. (2018). The comparative effectiveness and safety of three activated irrigation techniques in the isthmus area using a transparent tooth model. Int. Endod. J..

[47] Song J.S., Choi H.J., Jung I.Y., Jung H.S., Kim S.O. (2010). The prevalence and morphologic classification of distolingual roots in the mandibular molars in a Korean population. J. Endod..

[48] Pineda F., Kuttler Y. (1972). Mesiodistal and buccolingual roentgenographic investigation of 7275 root canals. Oral Surg. Oral Med. Oral Pathol..

